# The Strike in Lloyd's Tobacco Factory

**Published:** 1918-12-28

**Authors:** H. G. Lloyd

**Affiliations:** Richard Lloyd & Sons (Branch of Cope Bros. & Co., Ltd.), *Director.*


### THE EDITOR'S LETTER-BOX.

[Correspondence on all subjects is invited, but we cannot in any way be responsible for the opinions expressed by our correspondents, who must give their name and address as a guarantee of good faith, but not necessarily for publication. Correspondents are reminded that brevity of style and conciseness of statement greatly facilitate early insertion.]

### The Strike in Lloyd's Tobacco Factory.

*To the Editor of* The Hospital.

Sir,—We are sorry to find, in your report of the strike which recently occurred at this factory, that you have not made the fact quite clear, that the two wounded soldiers had only recently been engaged, and in neither case were they in our employ previously. A very large number of our people joined H.M. Forces, and in each case they will be re-engaged on their discharge, should they so desire it. This also applies to wounded men.

We should also like to state that we already have some of our original workers who have returned to us wounded, and who are now in receipt of the full Union rate of pay.

Trusting you will be able to find room in your paper for another word on this subject.—Yours faithfully,

Richard Lloyd & Sons (Branch of Cope Bros. & Co., Ltd.),

December 16, 1918. H. G. Lloyd, *Director*.

* We publish the above letter because Messrs. Lloyd wish it, and that there may be no shadow of doubt of the intention of the paragraph referred to. That intention, and it seems to us clear, was to point out that the ill-advised action of Messrs. Lloyds' hands would make employers, however sympathetic to our wounded men, hesitate to give such men a chance with Messrs. Lloyds' example before them of the difficulties with their employés such action may entail.—Ed. The Hospital.]

